# Vessel Detection and Tracking Method Based on Video Surveillance

**DOI:** 10.3390/s19235230

**Published:** 2019-11-28

**Authors:** Natalia Wawrzyniak, Tomasz Hyla, Adrian Popik

**Affiliations:** 1 Faculty of Navigation, Maritime University of Szczecin, 70-500 Szczecin, Poland; 2 Faculty of Computer Science and Information Technology, West Pomeranian University of Technology in Szczecin, 70-210 Szczecin, Poland; thyla@zut.edu.pl; 3 Marine Technology Ltd., 81-521 Gdynia, Poland; a.popik@marinetechnology.pl

**Keywords:** vessel detection, video monitoring, inland waterway, real-time detection

## Abstract

Ship detection and tracking is a basic task in any vessel traffic monitored area, whether marine or inland. It has a major impact on navigational safety and thus different systems and technologies are used to determine the best possible methods of detecting and identifying sailing units. Video monitoring is present in almost all of them, but it is usually operated manually and is used as a backup system. This is because of the difficulties in implementing an efficient and universal automatic detection method that would work in quickly alternating environmental conditions for all kind of sailing units—from kayaks to seagoing merchant vessels. This paper presents a method that allows the detection and tracking of ships using the video streams of existing monitoring systems for ports and rivers. The method and the results of experiments on three sets of data using cameras with different characteristics, settings, and scene locations are presented. The experiments were carried out in variable light and weather conditions, and a wide range of unit types were used as detection objectives. The results confirm the usability of the proposed solution; however, some minor issues were encountered in the presence of ships wakes or highly unfavourable weather conditions.

## 1. Introduction

Video surveillance systems are typically used to monitor vessels’ movement on coastal and inland waterways—especially those with heavy traffic, complicated organisation or which are in direct proximity to ports. However, the most typical way to track ships is using radar or Automatic Identifications System (AIS) infrastructure [1,2]. In most cases, waterside video surveillance works as a support for one of existing vessel traffic monitoring systems that uses wide collection of sensors and subsystems to acquire and distribute information on current traffic to systems users. (1) Vessel Traffic Services (VTS) is a marine system for ports and harbors that collects data on current traffic and assists ships in decision-making on area covered by the system. (2) River Information Services (RIS) are implemented in European waterways and serve as an information center for all systems’ recipients (ships, port authorities, and ship-owners) [3]. The information on detected and identified vessels in RIS can be pushed to other interconnected systems of administration (customs, police, etc.). (3) Integrated waterside security systems are developed in sensitive areas (port, naval bases, power plants, etc.) [4]. These systems detect and identify intruders using and fusing information from both underwater and above water with the use of sonars, echosounders [5,6] and autonomous vehicles [7]. In all of these systems, video surveillance helps to visually confirm the identification of a vessel or to monitor non-conventional (according to the International Convention for the Safety of Life at Sea (SOLAS)) units that are not obliged to be equipped with AIS transponders. Also, video monitoring is often used as a backup system and is seen as more reliable as a passive sensor. Currently, in most cases, a systems operator must visually monitor vessels that are passing in front of the cameras, particularly when it is necessary to detect and identify a wide range of vessels, which range from large cargo vessels to motorboats. Implementing detection and tracking algorithms to analyse live video streams allows for later automatic classification and identification of ships that enter or leave ports or other areas that need traffic support.

**Figure 1:**
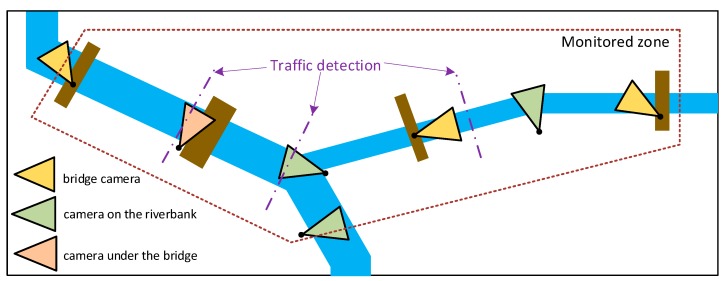
Vessel monitoring on a river based on video surveillance.

A video monitoring system can be used to track the status of all vessels that are present in a monitored zone. Especially, when the zone borders are established on rivers or canals. Additionally, each camera, that has a view from one bank to another, can be used to update vessels’ status or to count vessels passing a certain point on the waterway. The cameras can be placed in several different positions (Figure 1). The best view for the camera is usually from a bridge as passage under a bridge is often narrower than a waterway and zoom is not required to obtain a high-resolution image of a vessel. When a waterway is wide (hundreds of meters), a set of cameras is required to detect and identify passing vessels.

The automatic detection of vessels based on video stream analysis is difficult, mainly because the scene condition is constantly changing; e.g., the lighting conditions can be very dynamic due to sun reflections and the waves generated by wind and passing vessels. This causes difficulties in separating moving objects from the background. Generally, the object in a video stream can be detected using two basic solutions. The first solution is based on a pixel-based detection method that allows the detection of any moving object on a constant or slightly changing background. The second solution is object-based detection using a classifier; this second solution is usually used when it is possible to find a distinctive property of a class of objects—e.g., a specific type of prow.

Tracking vessels passing in front of the camera requires matching vessels that were detected in different video frames or using one of the standard tracking algorithms. The second approach is used when it is easy to specify the area of camera view in which objects enter and leave the scene. The tracking algorithms are generally faster than detection algorithms. Therefore, the choice of a tracking approach depends largely on performance requirements.

### 1.1. Related Works

Several researchers have proposed methods for object detection and tracking in video streams that did not assume a constant background, but were not specially dedicated for waterside systems. Their work was brought up and thoroughly compared in recent literature [8,9,10]. However, these solutions are usually too slow for use in real-time systems. Recent trend in object tracking and detection is to exploit the machine learning and pattern recognition methods; but again, the computational complexity of these solutions is high [11,12]. There are much fewer methods developed precisely for marine or costal environment with different approaches to detect ships. 

Ferreira et al. [13] proposed a solution for the vessel plate number identification of fishing vessels that enter and leave the harbor with the use of two cameras. The filming camera, with a low resolution, detects movements, and the photographic camera, with a high resolution, takes a photo when a movement is detected. In this solution, the type of the vessel is determined using object-based detection that looks for the prow of the vessel. Object-based detection is mainly based on a Histogram of Oriented Gradients (HOG) classifier [14]. 

In contrary, a pixel-based detection was used by Hu et al. [15] in a video surveillance system designed to detect and track intruder vessels approaching a cage aquaculture. They used the median scheme to create a background image from previous *N* frames and used a two-stage procedure to remove wave ripples. In the first stage, they used brightness and chromatic distortion to select some wave candidates, and in the second stage, they used brightness variation to finally select waves. 

In 2010, Szpak and Tapamo [16] also proposed a solution to the problem of vessel detection in the presence of waves. They used a background subtraction method and a real-time approximation of level-set-based curve evolution to distinguish moving vessels’ outlines. Another approach to improve detection (tracking) quality, based on a fusion of Bayesian and Kalman filters and the adaptive tracking algorithm, was proposed by Kim et al. [17]. Also, Kaido et al. [18] in 2016 proposed a two-stage method for detecting and tracking vessels based on edge selection and the support vector machine in the detection stage, and on a particle filter based on a colour histogram in the tracking stage.

In 2014, Moreira et al. [19] reviewed state-of-the-art algorithms related to the detection and tracking of maritime vessels. They concluded, among other results, that detection and tracking algorithms do not produce efficient results when applied to a maritime environment without proper adjustments; especially algorithms that have problems in real situations such as with small vessels that are hard to distinguish from the background due to low contrast.

There is also dynamic ongoing research into the methods used for the satellite optical imagery that is used to detect vessels. In 2010, Corbane et al. [20] proposed an operational vessel detection algorithm using high spatial resolution optical imagery. The algorithm is based on statistical methods and signal processing techniques (wavelet analysis, Radon transform). Another method based on shape and texture features was presented by Zhu et al. [21]. Later in 2013, Yang et al. [22] proposed another detection method based on sea surface analysis. More detection methods based on satellite imagery were published in [23,24,25].

In recent years, significant progress has been made in background/foreground segmentation algorithms that allow the detection of a moving object in the presence of a changing background. The background subtraction algorithms were evaluated in [26] and compared in [27]. Several background subtraction algorithms are implemented in the OpenCV library [28]. To begin with, the Gaussian Mixture-based Background–Foreground Segmentation (MOG) algorithm uses a mixture of three to five Gaussian distributions to model each background picture. The probable values of background pixels are the ones that are more static and present in most of the previous frames [29]. Next, Gaussian Mixture-based Background–Foreground Segmentation Algorithm version 2 (MOG2) [30] is available, which is an improved version of MOG. Other popular algorithms are the Godbehere–Matsukawa–Goldberg (GMG) method [31], which uses per-pixel Bayesian segmentation; CouNT (CNT), designed by Zeevi [32], which is designed for variable outdoor lighting conditions; *k* Nearest Neighbours (KNN), which implements K-nearest neighbours background subtraction, as shown in [33]; the algorithm created during the Google Summer of Code (GSOC) [28]; and Background Subtraction using the Local SVD Binary Pattern (LSBP) [34].

### 1.2. Motivation and Contribution

This paper is a part of an ongoing research in the Vessel Recognition (SHREC) [35] project, which concerns the automatic recognition and identification of non-conventional vessels in areas covered either by River Information Services or Vessel Traffic Service systems. The detection method is a first step in the automatic vessel identification and classification process. 

The main contribution of this research is a new vessel detection and tracking method. The method (1) detects all kind of moving vessels; (2) works in variable lightning conditions; (3) tracks vessels, i.e., it assigns a unique identifier to each vessel passing in front of the camera; and (4) is designed to be efficient, i.e., it can process Full High-Definition (FHD) and 4K video streams using economically acceptable amount of server resources. Based on several observations related to vessels movement and size characteristic, several rules were created that make it possible to distinguish between vessels and other moving objects. These rules were incorporated into several steps of the proposed method. Therefore, the proposed vessel detection method uses less image processing operations than existing ones. Additionally, a simple water area detection algorithm is used that detects water based on number and length of edges in a bounding box containing a moving object. The method was implemented, and the results from the experiments are described, which confirm that the proposed method is good to use in practice.

The preliminary version of the method was published in [36]. In contrast to that version, the proposed method contains a tracking function, a status update algorithm that includes additional filtering, a simple water detection algorithm, and several other minor improvements. The main difference compared to other vessel detection methods is the use of more logical processing (object filtering, tracking) than image processing techniques. Such an approach allows us to improve processing speed.

### 1.3. Paper Organisation

The rest of this paper is organised as follows. Section 2 contains the description of a novel Vessel Detection Method. Section 3 presents our test environment, test application, and experimental data sets. The final section discusses the experiment results and provides conclusions concerning the practical implementation of the proposed method.

## 2. Materials and Methods

### 2.1. Vessel Detection Method

**Figure 2:**
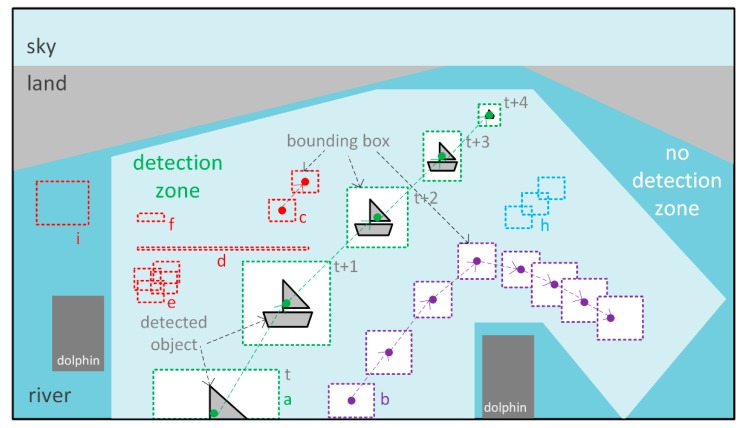
Schema of an exemplary video scene from a bridge camera with distinguished detection zone and different cases of bounding boxes being analysed in the method.

The proposed vessel detection method is designed using the following approach. The method assumes that for each camera view, there is a determined detection zone that eliminates areas of the scene where either ships cannot appear (e.g., on land) or they are too far for the detection process to make sense (Figure 2). The background subtraction algorithm is used for each frame from a video stream to obtain foreground objects, find their contours, and to obtain bounding boxes for these contours. These boxes are then used in analysis throughout the whole method. First, the bounding boxes outside of the detection zone (Figure 2i), boxes too small to be a vessel (Figure 2e,f), or with improper width to height ratio (Figure 2d) are removed. Next, the bounding boxes that contain water areas (e.g., waves, sun reflexes) are removed (Figure 2h). Then, the method matches the boxes from the current frame to boxes from previous frames based on several properties such as location or overlapping ratio and stores them in a buffer. Finally, once per every five frames, the boxes stored in the buffer that have movement features similar to passing vessels are returned as vessels (Figure 2a,b), while others are discarded (Figure 2c).

**Figure 3:**
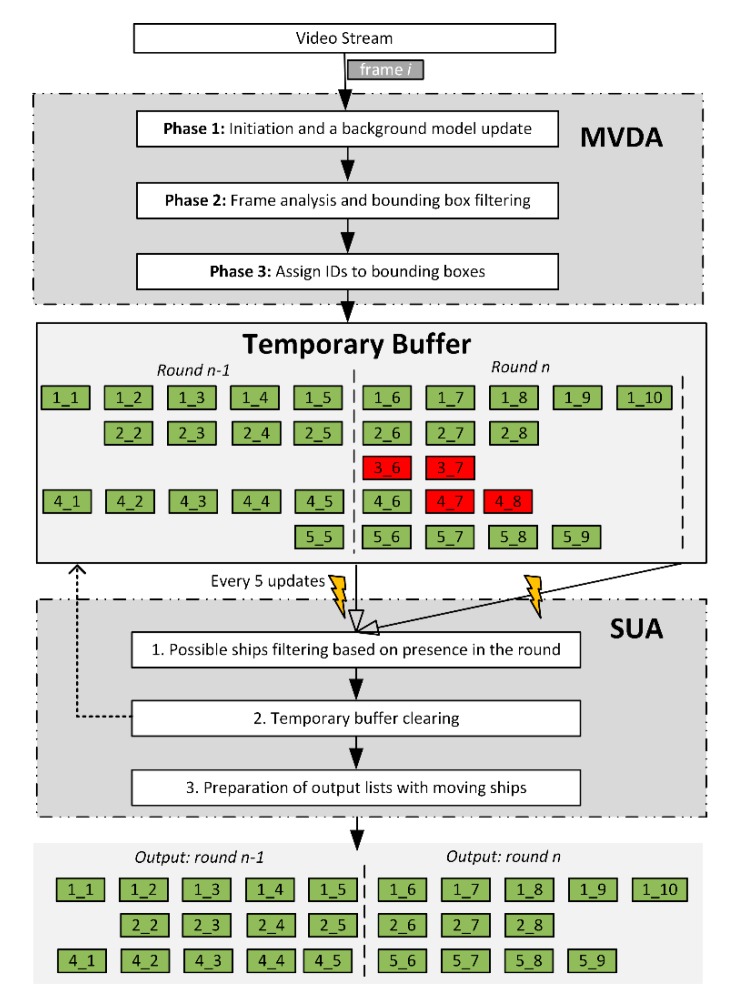
Vessel detection method. MVDA: Moving Vessel Detection Algorithm, SUA: Status Update Algorithm.

**Figure 4:**
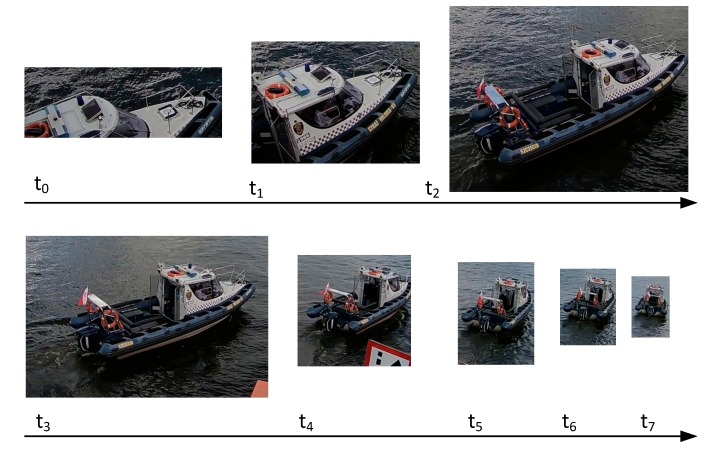
Example of MVDA output (a bridge camera—size corresponds to the size of the vessel in the video frames).

The vessel detection method consists of the Moving Vessel Detection Algorithm (MVDA), Status Update Algorithm (SUA), and Temporary Buffer (TB). The MVDA is responsible for returning bounding boxes with probable vessel locations for a given input video stream. The results are returned every 1 s and are stored in TB (Figure 3). At the end of every round (every 5 s), the SUA algorithm is run. The SUA inspects the content of TB, filters out probable artefacts, and returns 0 or more series of pictures containing the detected moving vessel. The example MVDA output for one passing vessel is presented in Figure 4.

### 2.2. Moving Vessel Detection Algorithm

The MVDA takes as an input a frame from a video stream and returns one or more bounding boxes with possible vessel locations. The algorithm works in three phases. In the first phase, the background model is initiated. In the second phase, a frame from a video stream is analysed, which results in a set of bounding boxes with probable locations of vessels in the frame. In the third and final phase, the algorithm assigns an identification number to each bounding box.

The detailed Algorithm 1 is as follows:

**Algorithm 1** Moving Vessel Detection Algorithm
1: **Phase 1**—Initiate a background subtractor (GSOC algorithm from OpenCV (Open Source BSD Licence):2:   For the first 250 frames, convert each frame to 720 p resolution, convert to grayscale and blur;3:   Update the background model with the result from step 1.1.4: **Phase 2**—Frame analysis of the video stream:5:   Convert the frame to 720 p resolution, convert to grayscale and blur;6:   If the frame is a multiple of 10, update the background model using it (for a stream of 30 frames per second; when streams have a different framerate, update the model using 3 frames per second).7:   For 1 frame per second, continue; otherwise, go to the analysis of the next frame;8:   Download the current background mask from the background model;9:   Search for edges in the background mask;10:   For each edge:11:      Calculate the bounding box;12:       Check the following conditions; if any of them is not met, proceed to the next edge analysis:13:            The bounding box is outside the negative area (the defined area on the scene for which no motion is detected);14:            The bounding box height is greater than the defined minimum value;15:            The bounding box width is greater than the defined minimum value;16:            The ratio of height to width of the envelope is less than the defined value;17:            The bounding box area is greater than the defined minimum value;18:            Check that the bounding box does not contain water using the Water Detection Algorithm (this algorithm detects the water area by checking the number and length of the edges).19:  Merge overlapping bounding boxes.20: **Phase 3**—Write the bounding box list into the Temporary Buffer:21:  For each bounding box:22:      Calculate the percentage of intersection of the bounding box with the bounding box detected in the previous frame;23:      Calculate the distance between the centre of the box and the boxes from the previous round;24:      Calculate the angle between the centre of the box and the boxes from the previous round;25:      Assign the current bounding box the ID number of the bounding box from the previous round with which the intersection area is the largest—if there is no such area, select the bounding box at a distance not greater than 1.5 from the current bounding box and with an angle difference not greater than the maximum value;26:      If the previous step failed to obtain the ID number, then create a new one;27:      Write the bounding box in the Temporary Buffer with the ID number.


### 2.3. Status Update Algorithm

The SUA’s goal is to eliminate any reaming artefacts. The algorithm is run every 5 s. It uses as its input data from the *Temporary Buffer.* It outputs 0 or more moving vessels; i.e., their pictures and movement direction. 

The detailed Algorithm 2 is as follows:

**Algorithm 2** Status Update Algorithm
1: For each set of bounding boxes with a given ID number that was present in the last 5 updates of the buffer (in the same round):2:      Verify if the set contains at least 3 bounding boxes in the round;3:      Verify if the average overlapping coefficient of the boxes’ areas for the set is greater than 0.2;4:      Verify if the average change of angle between the bounding boxes in the set is less than 60 degrees;5:      Verify if the centre of the bounding boxes is moving;6:      If one or more of the above conditions are not met, discard the set in this round;7:      Find the direction of movement of the vessel using the camera position information;8:      Cut the vessels’ pictures from the original video stream using a set of bounding boxes (requires bounding box scaling to the original stream resolution);9:      Create the output list with the pictures from the previous step—add the ID number and direction of movement to the output list; and 10:    Add the output list to the output list set.11: If the vessel with the ID number has not been updated by the last 5 updates of the buffer, remove it from the buffer.12: Return a set of output lists.


### 2.4. Water Detection Algorithm

The Water Detection Algorithm is based on an observation that all moving vessels have some kind of edges in contrast to water areas. The only situation when this algorithm is not able to detect water are waves behind moving vessels that have a lot of edges. In such a case, the algorithm is not able to differentiate them from vessels characteristics.

The Algorithm 3 is as follows:

**Algorithm 3** Water Detection Algorithm
1: Convert an image to grayscale, use bilateral filter, and blur the image;2: Detect edges using Canny edge detector and find contours;3: For each contour:4:      Calculate length of the contour (using curve approximation);5:      Calculate the contour area;6:      When the length is less than 100 or the area is less than 30, discard the contour;7: Calculate average length of the remaining contours and find the longest contour;8: Return water when number of contours is less than 3, the maximum length is less than 250, and average length is less than 40.


## 3. Results

### 3.1. Test Environment

**Figure 5:**
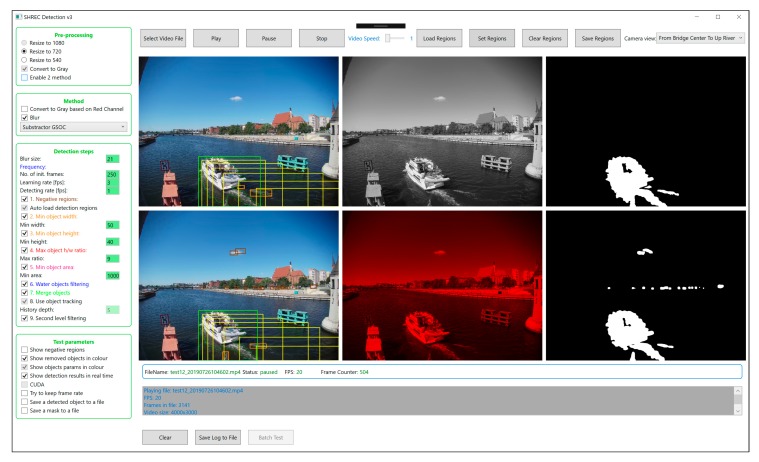
A screenshot from the test application.

The method was implemented using C# (Microsoft Corporation, Redmond, Washington, DC, USa) and Emgu CV version 4.0.1 (EMGU Corporation, Markham, Ontario, Canada) (C# wrapper for OpenCV). The application (Figure 5) allows the user to select different parameters and see how they affect the detection quality. It is possible to observe the detection performed by two methods simultaneously to allow for better comparison. Some of the intermediate results are visualised to help us to better understand the impact of each element of the method on the final result. Additionally, it is possible to run a batch test for a given set of video files and store the detection results into the files.

Three data sets were used to test the quality of the proposed detection method:

1. Data Set A—the preliminary set, containing 15 different video samples (Full High Definition 1920 × 1080, 30 fps, bitrate: 20 Mb/s, AVC Baseline@L4, duration: between 30 s and 120 s), which shows different types of vessels from different angles, recorded in different places using GoPro Hero 6 cameras (GoPro Inc., San Mateo, CA, USA) The set was also used to test the first version of our method [24].
2. Data Set B—the set containing 20 video samples (4000 × 3000, 20 fps, bitrate 8 Mb/s, AVC High@L5.1, duration: between 27 s and 434 s) recorded using an AXIS IP camera Q1765-LE 1080 p motozoom AXIS (Axis Communications AB, Lund, Sweden)
3. Data Set C—the set containing 20 video samples (Full High Definition 1920 × 1080, 25 fps, bitrate: 2–7 Mb/s, AVC Main@L4.1, duration: between 48 s and 224 s) recorded using a Dahua IP camera IPC-HFW81230E-ZEH 12Mpx (Dahua Technology Co., Ltd., Hangzhou, China).

**Figure 6:**
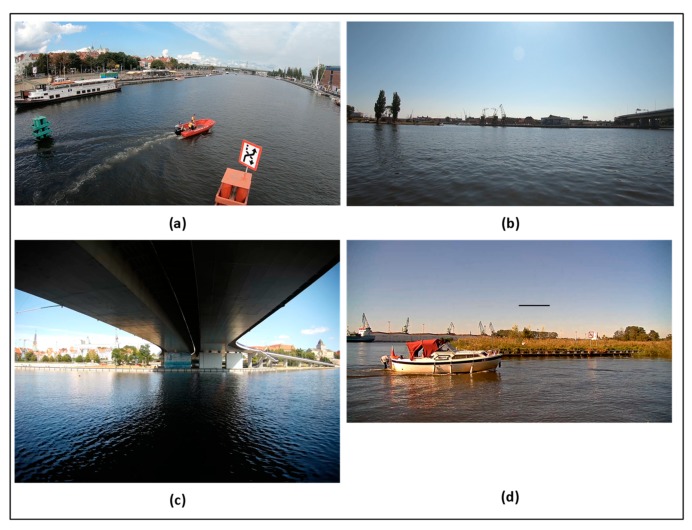
A screenshot from different video samples from sets A, B, and C: (**a**) bridge view; (**b**) bank view–close perspective; (**c**) under bridge view; (**d**) bank view–distant perspective.

Figure 6 presents sample (not all) camera views from sets A–C. View (a) is the view from the center of the bridge, view (b) is the view from the river bank, view (c) is the view from below a high suspended bridge, and view (d) is a canal bank.

Two sets of settings were used in the tests:

- Standards settings: blur_size = 21, use_red_channel = false, min_width = 50, min_height = 40, max_ratio = 9, min_area = 2500, water_sensitivity = normal;
- High threshold settings: blur_size = 21, use_red_channel = false, min_width = 65, min_height = 50, max_ratio = 10, min_area = 5000, water_sensitivity = high;

The method was tested using a test computer (Core i7-8700K (Intel Corporation, Santa Clara, CA, USA)., 32GB RAM., SSD 1TB NVIDIA Quadro P4000 (Nvidia, Santa Clara, CA, USA)). Each test outputted a set of image files that were categorised to each catgory of detection events by two experts.

### 3.2. Experiments

The detection events were divided into the following categories:

1. Correct detection—a vessel was detected once per passage in front of the camera view, i.e., a series of pictures of the vessel with one identification number was returned;
2. Semi-correct detection Type I—a vessel was detected more than once per passage in front of the camera view, i.e., two or more series of pictures of one vessel with different idetification numbers was returned;
3. Semi-correct detection Type II—a vessel was detected once per passage in front of the camera view, but some pictures in the series contained small artefacts;
4. Incorrect detection Type I—a vessel was not detected;
5. Incorrect detection Type IIa—a series of artefacts were returned incorrectly as a vessel; in this category, all artefacts exluding water are included;
6. Incorrect detection Type IIb—a series of artefacts were returned incorrectly as a vessel; in this category, all water artefacts are included.

The semi-correct results can be later corrected by the final vessel identification algorithm, which is not a part of the detection method. For example, when a series containing 20 vessel images contains a water artefact in the end, this is not a problem, as the identification algorithm does not use boundary images.

**Figure 7:**
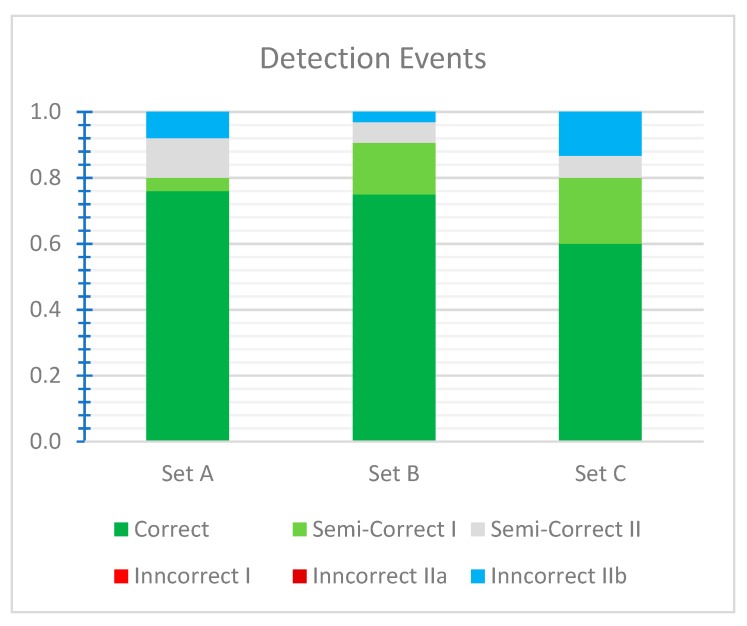
Detection events for datasets A, B, and C and standard settings.

The results for datasets A, B, and C and standard settings are presented in Figure 7. The method returned around 75% of correct detection events for sets A and B and 60% for set C. The semi-correct detection events accounted for 16% of the total events for set A, 24% for set B, and 27% for set C. The results from set A contain more Type II semi-correct events, and those from set B and C exhibit more Type I semi-correct events. For all sets, there were no inncorrect detection Type I and IIa events; i.e., all vessels were detected, and series containing only artefacts were not returned. The only incorrect results are Type IIb events, which are series of pictures with water-only artefacts.

**Figure 8:**
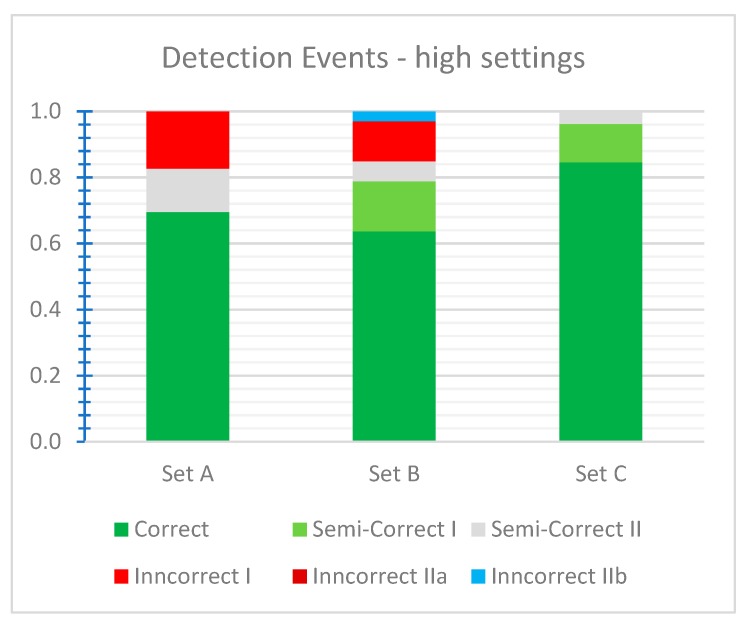
Detection events for datasets A, B, and C and high-threshold settings.

The results for high-threshold settings are presented in Figure 8. In contrast to standard settings, the incorrect detection events of type I (the vessel was not detected) are presents for sets A and B. Also, water artefacts are not present in sets A and C. The method with high settings returned more correct results for set C (85%) and fewer for sets A (70%) and B (64%), mainly because these sets contain more small vessels that were filtered out.

## 4. Discussion

The incorrect detection events mainly arose from a few video samples with unfavourable lightning conditions. The video samples that come from a camera facing the sun have less colour saturation, and sun reflexes are present. This causes the background subtraction algorithm to provide worse results. Another difficult case is of a camera placed below a high suspended bridge (Figure 6c) that has a large shadow in the middle of the camera view. In practical deployment, such situations can be avoided in most cases by carefully placing surveillance cameras; for example, the cameras can be placed on the north side of the bridge (on northern hemisphere) or before it to avoid such cases.

The method for the input resizes the input frames to 1280 × 720 resolution. This resolution is a trade-off between processing speed and accuracy. Set B, despite the fact that it has higher resolution, is more compressed and has visible compression artefacts. This means that, after downsizing the frame, the picture has fewer sharp edges. The method uses a blurred frame for background–foreground separation, but a non-blurred frame is used for water artefact filtering. Because of that, water artefacts were better filtered in set B, as water detection is based mostly on counting edges in the image.

It is worthwhile to note that the professional camera used to obtain datasets B and C produced lower quality images than our GoPro camera. This is mainly because video samples were recorded locally on an memory card. The video samples from the other two cameras were recorded from the video streams coming from these cameras with the highest possible quality settings.

One of the main problems in vessel detection are waves. However, the waves in inland waters caused by wind are significantly smaller than waves at sea. In the evaluated datasets, the proposed method removes all these waves. The few water artefacts that are returned (Type IIb incorrect detection events) are caused by the wakes created by a moving vessel. These water artefacts have more small sharp edges than small boats and therefore are not eliminated. This can be changed by setting the sensitivity to a higher threshold in the water detection algorithm. The output from the proposed method is further used in the vessel classification method based on deep neural networks (DNN). One of the defined classes is water, so that it can be later eliminated. The only reason that we do not use DNN in the MVDA is efficiency, as our tests showed that using DNN in the detection method takes too much time.

The results are not uniformly distributed among samples from different camera views. Samples from camera views with sunlight from behind and which are placed on the centre of the bridge or placed perpendicular to a narrow waterway provide practically no incorrect detection results.

The main limitation of the study is that the method is designed to work in daytime without large atmospheric precipitation. One of the other known limitations is the problem of distinguishing vessels in a situation where one big vessel is in front of the camera and small ones enter the camera view when the other vessel is in the background. In such cases, the method returns a frame with two vessels. In further steps in the final identification algorithm, it will be possible to detect hull inscriptions from both vessels, and in this way, the vessel can be distinguished.

Future works include improving the method by adjusting it to work with video streams obtained from infrared cameras at night and detecting vessels passing in proximity. Future improvements also include adding the ability to work in low-light conditions; for example, when the camera is placed on a bridge in a city during the night, when there is residual light from street lamps.
